# The role of Ag^+^, Ca^2+^, Pb^2+^ and Al^3+^ adions in the SERS turn-on effect of anionic analytes

**DOI:** 10.3762/bjnano.10.224

**Published:** 2019-11-27

**Authors:** Stefania D Iancu, Andrei Stefancu, Vlad Moisoiu, Loredana F Leopold, Nicolae Leopold

**Affiliations:** 1 Faculty of Physics, Babeș-Bolyai University, Kogalniceanu 1, 400084 Cluj-Napoca, Romania; 2 Faculty of Food Science and Technology, University of Agricultural Sciences and Veterinary Medicine, Manastur 3-5, 400372 Cluj-Napoca, Romania

**Keywords:** adion-specific adsorption model, cation bridging, Raman, surface enhanced Raman scattering (SERS)

## Abstract

In our recent studies we highlighted the role of adsorbed ions (adions) in turning on the surface-enhanced Raman scattering (SERS) effect in a specific mode for anionic and cationic analytes. In this work, we emphasize the role of Ag^+^, Ca^2+^, Pb^2+^ and Al^3+^ adions in the specific adsorption of anionic analytes such as the citrate capping agent and three organic acids. Our results suggest an adion-specific adsorption mechanism: the adsorption of anionic analytes is facilitated by positively charged adions such as Ag^+^, Ca^2+^, Pb^2+^ or Al^3+^, which provide adsorption sites specific for the anionic analytes. The turn-on of the SERS effect is explained in the context of the chemical mechanism of SERS. The adions form SERS-active sites on the silver surface enabling a charge transfer between the adsorbate and the silver surface. High-intensity SERS spectra of uric acid, salicylic acid and fumaric acid could be recorded at a concentration of 50 µM only after activation of the colloidal silver nanoparticles by Ca^2+^, Pb^2+^ or Al^3+^ (50 µM). The chemisorption of the three anionic species to the silver surface occurs competitively and is enhanced with the anions of higher affinities to the silver surface as indicated by the SERS spectra of corresponding mixed solutions.

## Introduction

Surface-enhanced Raman scattering (SERS) is an ultrasensitive technique with detection limits below nanomolar concentrations and is able to resolve single molecules of cationic dyes such as rhodamine 6G or crystal violet. The SERS detection of such cationic analytes has been achieved by using chloride-activated colloids, with chloride assuming the role of an aggregation agent [1–2]. Consequently, many studies explain the SERS effect by the formation of electromagnetic hot-spots, i.e., sites with highly increased field strengths generated by the aggregated nanoparticles.

Early SERS studies highlighted the importance of a strong electronic coupling between the adsorbate and the metal nanosurface, the coupling to the silver surface being mediated by adsorbed atoms (adatoms) such as Ag^+^, Cl^−^, I^−^, Br^−^ [3–6]. In this context, several reports explain the SERS enhancement by the formation of stable surface complexes of atomic scale roughness. For example, a Ag^+^–halide–organic molecule is formed that allows a charge transfer between the metal surface and the molecule leading to a resonant Raman scattering effect [6–8].

Evidence for surface complexes were provided by several SERS experiments on silver electrodes [3,8], but also on colloidal silver nanoparticles (AgNPs) [9–11]. Muniz-Miranda and Sbrana showed that Ag^+^ adsorbed ions (adions) can be generated on a metallic surface by co-adsorbed nucleophilic anions (such as Cl^−^, I^−^, Br^−^, SCN^−^) leading to the formation of Ag^+^–phtalazine–anion complexes on the surface of AgNPs [9].

The role of Ag^+^ adions in the SERS effect was evidenced experimentally also by Watanabe et al. and Doering et al., who showed that thiosulfate, a photographic fixing agent that dissolves Ag^+^ ions, completely quenches the SERS signal [8,12]. Furthermore, the aggregation of nanoparticles was completely excluded since the AgNPs were immobilized on a glass surface [12].

Recently, we brought further evidence for the role of the Cl^−^ adion in the SERS detection of cationic molecules, showing that Cl^−^ (but also Br^−^ or I^−^) adions promote the chemisorption of cationic dyes to the silver surface leading to the turn-on of the SERS effect for cationic molecules [13–14]. Moreover, we showed in our studies that Ag^+^, Ca^2+^ or Mg^2+^ adions promote the chemisorption of Cl^−^, thus forming specific SERS-active sites for cationic molecules such as rhodamine 6G at 10^−11^ M or nile blue at 10^−8^ M. In this low-concentration regime, we were able to detect the SERS signal of the cationic dyes only after facilitating the chemisorption of Cl^−^ ions by Ag^+^, Ca^2+^ or Mg^2+^ adions [13–14]. We explained this surface process by an adion-specific adsorption model and the SERS enhancement in the context of the electronic mechanism of SERS, which state that the Raman enhancement appears due to the electronic analyte–metal surface coupling mediated by the adions, which play the role of SERS-active sites [13–14].

In the proposed adion-specific adsorption model, adions present on the surface of the nanoparticles lead to an ion-specific effect: adsorbed cations such as Ag^+^, Mg^2+^ and Ca^2+^ promote the chemisorption of anionic species including Cl^−^, Br^−^ and I^−^ making possible their observation by SERS. Likewise, the Cl^−^, Br^−^ and I^−^ adions mediate the chemisorption of cationic analytes switching on their SERS spectrum [13–14]. This surface activation strategy greatly improves the selectivity of SERS enabling the detection of target analytes even from complex biological matrixes [13–14].

In this study, we show that the SERS spectra of organic acids such as uric, salicylic or fumaric acid, but also the SERS spectrum of the citrate anion capping agent, are observed in the presence of adsorbed cations such as Ca^2+^, Pb^2+^, Al^3+^ or Ag^+^. These adions form SERS-active sites on the surface of the nanoparticles facilitating the chemisorption of anionic analytes and the switch-on of their SERS spectra. This surface activation strategy is promising for improving the reliability of SERS detection for anionic target molecules and also for improving the selectivity of SERS detection.

## Results and Discussion

SERS studies on anionic analytes are considerably fewer in the literature than studies on cationic molecules. The competitive adsorption of anionic species to the nanoparticle surface makes their SERS detection challenging since the most used SERS colloids contain anionic capping agents such as chloride or citrate. Table 1 summarizes several SERS studies of anionic species showing the detected anionic analyte, the concentration and the employed SERS substrate.

**Table 1:** SERS studies of anionic analytes.

anionic analyte (concentration)	SERS substrate
	
cyanide (10 mM)	gold electrode [15]
picric acid (0.36 mM), diclofenac (0.05 mM)	thiocoline modified colloidal AgNPs prepared by hydroxylamine hydrochloride reduction [16]
malonic acid (1 mM), oxalic acid (1 mM), succinic acid (1 mM)	colloidal AgNPs obtained by borohydride reduction [17]
benzoic acid (2 mM)	colloidal AgNPs obtained by hydrogen peroxide reduction [18]
dipicolinic acid (0.02 mM)	colloidal AgNPs obtained by citrate reduction [19]
bilirubin (1 nM)	poly-ʟ-lysine-coated AgNPs obtained by citrate reduction [20]
gallic acid (1 mM)	dried AgNPs prepared by hydroxylamine hydrochloride reduction [21]
hydoxybenzoic acid (1 mM)	colloidal AgNPs obtained by borohydride reduction [22]
salicylic acid (0.1 mM)	colloidal AgNPs obtained by borohydride or citrate reduction [23]
uric acid (0.25 mM)	colloidal AgNPs obtained by or hydroxylamine hydrochloride reduction [24]

It is clear from Table 1 that most studies regarding SERS detection of anionic analytes are reported in the millimolar concentration range. Particularly, the SERS spectrum of bilirubin [20] could be obtained at nanomolar concentration due to the resonance Raman supplementary enhancement mechanism. In the present study, we show that SERS spectra of anionic analytes can be obtained in the micromolar concentration range, by activating the AgNPs with cationic adions.

### Switch-on of the citrate capping agent SERS spectrum

For this study we chose citrate-capped silver nanoparticles (cit-AgNPs) since they provide a good trade-off between colloidal stability and surfactant affinity to the AgNP. We avoided the use of chloride-containing silver colloids [13,25] because of the competitive chemisorption between the anionic analyte and the Cl^−^ capping agent to the Ca^2+^, Pb^2+^ or Al^3+^ activated AgNPs, Cl^−^ showing a high affinity for the silver surface. The colloidal nanoparticles used here are surrounded by citrate anions in an electrostatic interaction with the silver surface, which confer the nanoparticles a negative zeta potential and thus electrostatically stabilize the AgNPs and prevent aggregation.

In our previous reports, we showed that the SERS spectrum of the citrate capping agent can be switched on by the addition of cations such as Ag^+^, Ca^2+^ or Mg^2+^, the chemisorption of citrate to silver surface being mediated by these adions [13,26]. In this study, we show that the SERS spectrum of the citrate capping agent can be switched on by the intrinsic generation of Ag^+^ ions at the AgNP surface.

**Figure 1:**
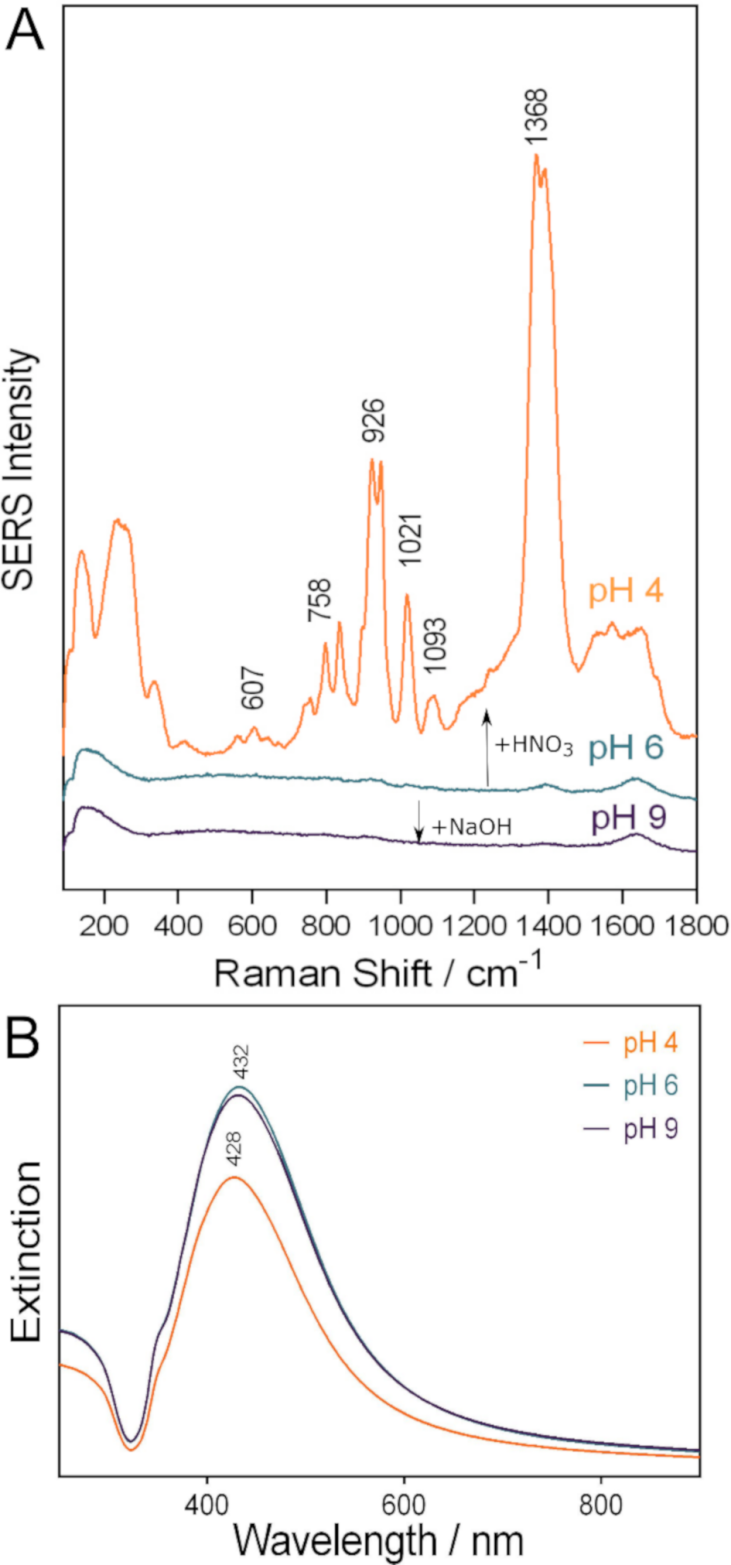
(A) SERS spectra and (B) UV–vis extinction spectra of the colloidal solution of cit-AgNPs at pH values between 4 and 9 as indicated in the figure.

The addition of HNO_3_ to the colloidal cit-AgNPs leads to the generation of Ag^+^ ions at the silver surface due to an incipient dissolution process of the AgNPs in the acidic medium [27–28]. Thus, by lowering the pH value of the cit-AgNP solution from 6 to 4, citrate chemisorbs to the AgNPs owing to the Ag^+^ SERS-active sites formed on the AgNP surface. As a result, the SERS spectrum of citrate is switched on (Figure 1). We recorded a SERS spectrum of citrate of similar shape previously by supplementing the cit-AgNPs with 1 mM AgNO_3_, which generated additional Ag^+^ SERS-active sites on the nanoparticle surface [13].

However, the adsorption affinity of citrate to the Ag^+^ activated silver surface is ≈10 times lower than that to the Ca^2+^ or Mg^2+^ activated silver surface [13]. Therefore, an additional increase in the intensity of the SERS spectrum of citrate recorded from the cit-AgNP solution at pH 4 is observed after the addition of 50 µM Al^3+^ (Supporting Information File 1, Figure S1). This effect is due to the additional SERS-active sites formed by the Al^3+^ adions on the AgNP surface. Moreover, a higher affinity of the citrate anions to the Al^3+^ activated surface than to the Ag^+^ activated surface is supposed. As further shown, the Al^3+^ active sites enable the recording of high-intensity SERS spectra for all three organic acids employed in this study.

The aggregation of the silver colloids at pH 4 was excluded since no broadening or any additional extinction bands at higher wavelengths were observed in the corresponding UV–vis spectrum (Figure 1B). The incipient dissolution of the AgNPs and the formation of Ag^+^ ions on the surface of the nanoparticles leads to the adsorption of citrate to the surface of the AgNPs. Therefore, the observed blue shift and damping of the surface plasmon resonance (SPR) peak, which is observed only after the formation of Ag^+^ adions, indicates an electronic contact between the AgNPs and citrate (Figure 1B) [28–30].

No SERS spectra of citrate were obtained at pH 6 and pH 9. Instead, the recorded spectral shapes are similar to the Raman spectrum of water (Figure 1A).

### SERS of organic acids mediated by Ca^2+^, Pb^2+^ or Al^3+^ adions

We aimed to show that the adsorbed Ca^2+^, Pb^2+^ or Al^3+^ cations lead to the chemisorption of organic acids enabling their SERS detection. We probed this methodology on three organic acids: uric acid, salicylic acid and fumaric acid.

Uric acid is a metabolite present in biofluids such as blood serum (0.2–0.4 mM) [31], urine or saliva. The SERS spectra of these biofluids are dominated by uric acid and other purine metabolite bands due to the high affinity of these metabolites for the silver surface. Because the affinity of uric acid to the AgNPs is higher than that of chloride, uric acid can be detected by SERS also with chloride containing colloids [24,26].

**Figure 2:**
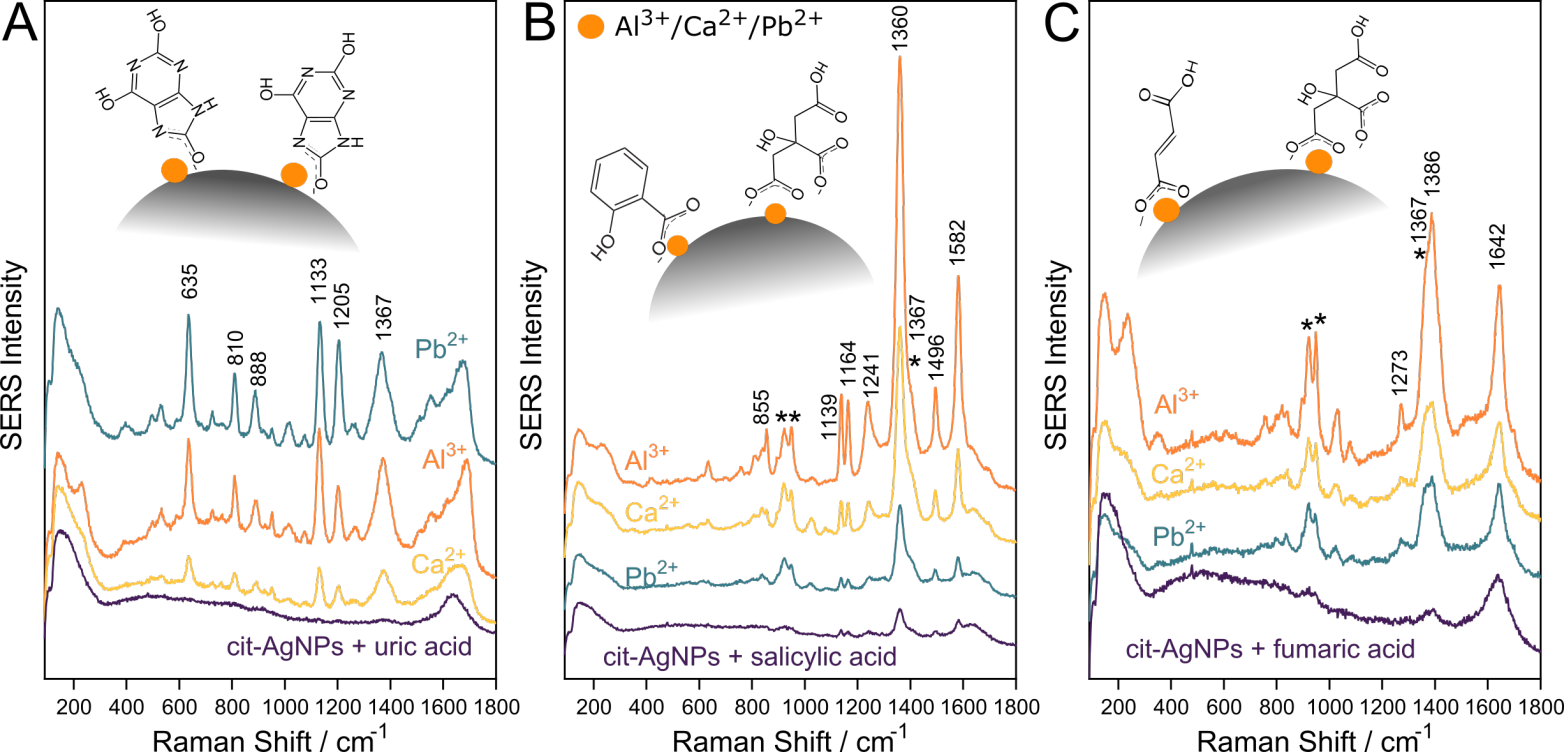
SERS spectra of 50 µM organic acids after their addition to cit-AgNPs and after the activation of the AgNPs with 50 µM Ca^2+^, Pb^2+^ or Al^3+^, as indicated in the figure: (A) uric acid, (B) salicylic acid and (C) fumaric acid. The insets show the proposed chemisorption geometry of the anionic analytes on the silver surface. Contributions to the spectra from the citrate capping agent are marked by asterisk.

However, at low concentrations such as 50 µM, neither uric acid nor fumaric acid exhibit SERS spectra when probed with as synthesized cit-AgNPs as the substrate (Figure 2A and C). Only in the case of salicylic acid a very weak SERS signal is observed (Figure 2B).

The addition of cations such as Ca^2+^, Pb^2+^ or Al^3+^ at a final concentration of 50 µM to the cit-AgNPs/organic acid mixture allows one to obtain intense SERS signals from the three organic acids at a concentration of 50 µM. Figure 2 presents the SERS spectra of uric acid, salicylic acid and fumaric acid with cit-AgNPs as substrate recorded at pH 6 before and after the addition of Ca^2+^, Pb^2+^ or Al^3+^ to the colloidal solution.

The Raman enhancement is not a result of the colloid aggregation since no broadening of the SPR band was observed in the colloid extinction spectra after the SERS activation of the cit-AgNPs/organic acid mixtures with cations. A weak damping of the SPR band is observed only after the addition of cations that facilitate the chemisorption of the organic acids to the silver surface (Supporting Information File 1, Figure S2).

Figure 2A shows well defined uric acid SERS bands at 635, 810, 888, 1133, 1205 and 1367 cm^−1^ after activating the cit-AgNPs with Ca^2+^, Pb^2+^ or Al^3+^ adions [32]. In the spectrum, no citrate characteristic SERS bands are observed. The Al^3+^ activated SERS spectrum of uric acid and the Raman spectrum are shown in the Supporting Information File 1, Figure S3A.

The characteristic SERS bands of salicylic acid are observed at 855, 1139, 1164, 1241, 1360, 1496 and 1582 cm^−1^ (Figure 2B). Comparing the Raman spectrum of salicylic acid in aqueous solution (0.1 M) with the SERS spectrum reveals that several bands shift to lower wave numbers due to the interaction of the adsorbate with the silver surface (Supporting Information File 1, Figure S3B). For example, the C=C stretching vibration observed at 1587 cm^−1^ in the Raman spectrum shifts to 1582 cm^−1^ in the SERS spectrum.

The SERS spectrum of salicylic acid at a concentration of 0.1 mM was reported previously by Alvarez-Puebla and co-workers [23]. The SERS spectrum could be obtained only at acidic pH values of the colloidal cit-AgNP solution, but not at basic pH values. In this study, this pH limitation is overcome. By activating the colloid with 50 µM Al^3+^ the SERS spectra of salicylic acid could be obtained in the pH 6–9 range, as shown in Supporting Information File 1, Figure S4.

In the case of fumaric acid, the interaction with the silver surface induces even more pronounced spectral shifts. The C=C stretching vibration observed at 1653 cm^−1^ in the Raman spectrum of the 0.1 M solution shifts to 1642 cm^−1^ in the SERS spectrum (Supporting Information File 1, Figure S3C).

In the SERS spectra of salicylic acid and fumaric acid, the SERS bands of the citrate capping agent are observed at 924 and 949 cm^−1^, indicating a co-adsorption of citrate with the analytes. The shoulder at 1367 cm^−1^ (Figure 2B,C) also represents a contribution of citrate. This large SERS band is assigned to the in-phase R-CO_2_ stretching vibration, which appears usually as a strong band in the 1360–1450 cm^−1^ range of the Raman spectra of carboxylic acids [33–34].

It can be observed that for uric acid the highest Raman enhancement is obtained when the chemisorption is mediated by Pb^2+^ adions, while for salicylic and fumaric acid Al^3+^ leads to the highest Raman enhancement. The formation of adion–organic acid surface complexes is supposed, the chemisorption taking place via the carboxyl group in the case of salicylic acid and fumaric acid and via the NCO^−^ group in the case of uric acid. The proposed adsorption geometries of the three organic acids on the silver surface are shown in the insets of Figure 2.

### SERS selectivity – competitive adsorption to the silver surface

As the chemisorption of anionic species to the silver surface occurs competitively, anionic species with a higher affinity for the silver surface than citrate such as Cl^−^ will displace citrate from the surface of the nanoparticles, thereby switching-off the SERS signal of citrate [13,26].

**Figure 3:**
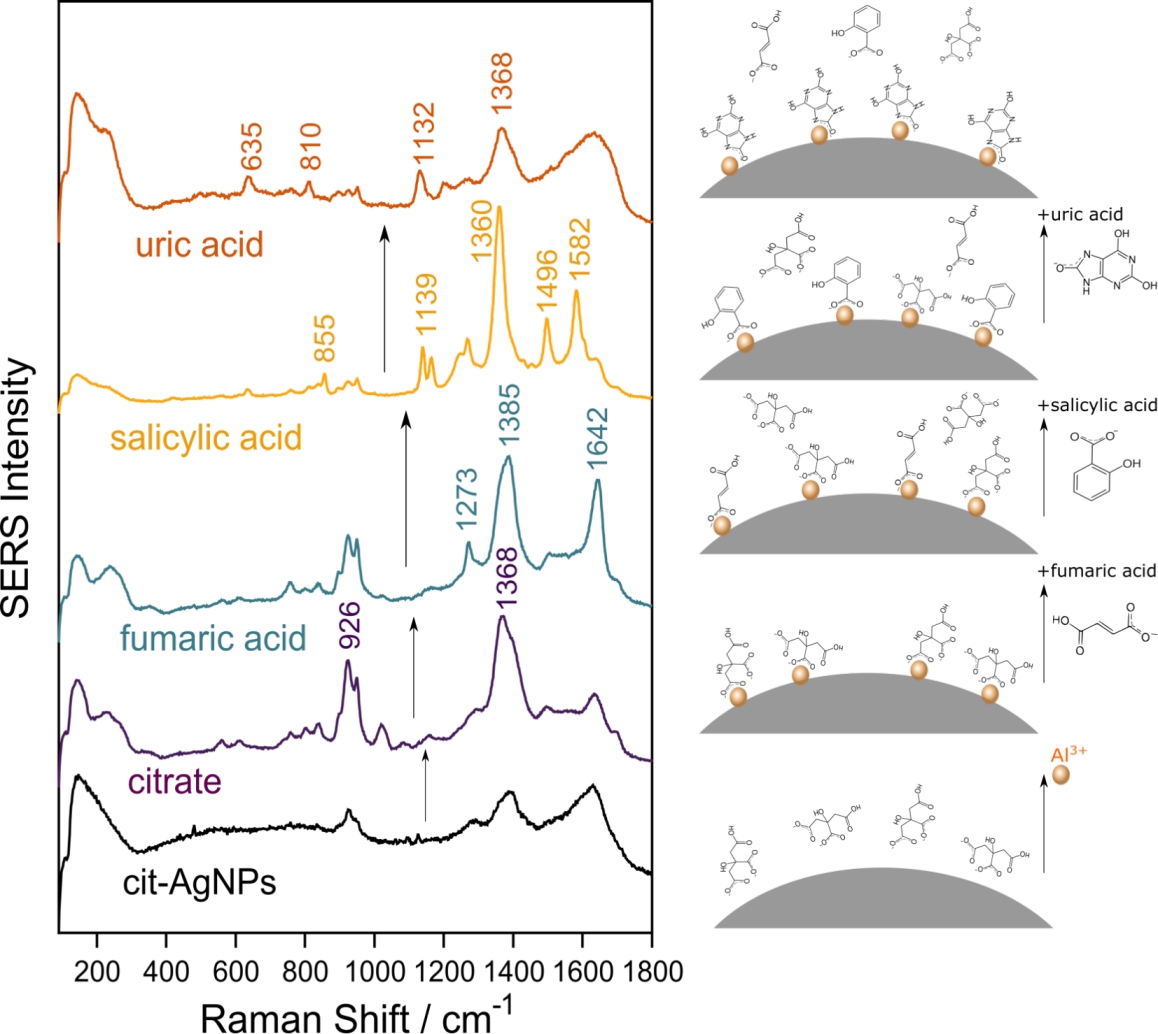
From bottom to top: SERS spectrum of the cit-AgNPs as synthesized, SERS spectrum of citrate after activation of the colloids with 50 µM Al^3+^, SERS spectra of the same solution with fumaric acid, salicylic acid and uric acid added subsequently. The SERS spectra were recorded by averaging four acquisitions of 10 s exposure each and were normalized to unity.

Figure 3 shows the selectively recorded SERS spectra of the organic acid mixtures. To record these, fumaric acid, salicylic acid and uric acid were added sequentially to the Al^3+^ activated cit-AgNP solution.

The three organic acids were added is in the order of increasing affinity to the silver surface. The cit-AgNPs probed as synthesized show weak SERS signals at 926 and 1370 cm^−1^ assigned to citrate. The addition of Al^3+^ ions to the cit-AgNP solution promotes the chemisorption of citrate to the AgNPs leading to high-intensity SERS bands of citrate.

By adding fumaric acid at a concentration of 50 µM to the same solution, new bands appear at 1642 and 1273 cm^−1^, which can be assigned to fumaric acid. The SERS bands of citrate are still evident indicating that fumaric acid co-adsorbs with citrate on the silver surface. However, the high-intensity of the band at 1380 cm^−1^ indicates a contribution of both anionic species. The presence of the SERS features of both anionic species, fumaric acid and citrate, indicates that both anions have comparative affinities for the Al^3+^ activated silver surface. However, it has to be mentioned that a much higher concentration of citrate is present in the colloidal solution 1.3 mM compared to 50 µM fumaric acid.

Next, salicylic acid at a concentration of 50 µM was added to the same mixture. As a result, at 855, 1139, 1360 1496 and 1582 cm^−1^ the SERS bands of salicylic acid were observed with high intensities. The SERS features of the previously adsorbed anions disappeared since both, citrate and fumaric acid, were displaced by salicylic acid on the silver surface.

However, the highest affinity to the silver surface is shown by uric acid. Once uric acid is added to the mixture, the intense SERS spectrum of salicylic acid disappears and instead the SERS features of uric acid are prominent at 635, 810, 1132 and 1368 cm^−1^.

### Adion-specific adsorption model

As shown in this study, we are able to detect the SERS signals of three anionic analytes at concentrations of 50 µM only after the activation of cit-AgNPs with Ag^+^, Ca^2+^, Pb^2+^ or Al^3+^ adions. The adions promote the specific chemisorption of the anionic analytes followed by a SERS turn-on effect. The proposed mechanism for the specific adsorption follows the electrosorption model on metallic surfaces presented by Attard, which is based on two postulates [35]:

1. anions will only adsorb on locally positively charged sites, and
2. cations will only adsorb on locally negatively charged sites.

Thus, in the study presented here, cations such as Ca^2+^, Pb^2+^ or Al^3+^ adsorb on available negatively charged sites on the surface of the cit-AgNPs such as surface defects and kinks [36–37] and will increase the number of available positively charged adsorption sites for anionic analytes.

As highlighted by Attard, the polarizability of the adion will influence greatly its surface activity [35]. This explains why cations with a high polarizability such as Ca^2+^, Pb^2+^ or Al^3+^ promote the specific adsorption of anionic analytes, whereas cations with a low polarizability such as Na^+^ have a negligible effect on the surface adsorption.

In order to provide further evidence in support of this model, we showed that only the cationic adions present on the silver surface are responsible for the specific adsorption of the anionic analytes enabling a SERS switch-on effect. For this, we eliminated the excess of free, unadsorbed Ca^2+^, Pb^2+^ or Al^3+^ from the colloidal solution by washing the activated cit-AgNPs as described in the Supporting Information File 1, Figure S5. Salicylic acid (50 µM) was added to the washed cit-AgNPs. The SERS spectrum of salicylic acid could be obtained only for the solutions containing cit-AgNPs that were activated with Ca^2+^, Pb^2+^ or Al^3+^ prior to washing (Supporting Information File 1, Figure S5), whereas for the solutions with cit-AgNPs that were not activated with cations prior to washing, we did not obtain any SERS signal of salicylic acid indicating that salicylic acid did not adsorb on the bare cit-AgNPs. These results confirm that only the adsorbed cations facilitate the chemisorption of the anionic analytes, while the free cations present in the solution do not influence the specific adsorption. These observations reinforce the results derived from Figure 2B, in which the SERS spectrum of salicylic acid was turned-on only by the adion-specific activated nanoparticles.

## Conclusion

The results presented in this study show that the chemisorption of anionic analytes to cit-AgNPs highly depends on both the chemical structure of the target analyte and the intrinsic properties of the nanoparticle surface, which dictate the interaction with the molecule.

The adsorption of citrate, uric acid, salicylic acid and fumaric acid can be explained by an adion-specific adsorption model. The SERS spectra of these analytes are obtained only after the activation of the cit-AgNPs with Ca^2+^, Pb^2+^ or Al^3+^ adions. The cationic adions promote the specific chemisorption of the anionic analytes, thus enabling a SERS turn-on effect. Similarly, the intrinsic generation of Ag^+^ adions by the partial dissolution of cit-AgNPs in an acidic environment gives rise to the switch-on of the SERS spectrum of the citrate capping agent.

The chemisorption of the target analytes to the cit-AgNPs can be monitored qualitatively through the damping of the SPR band. Particularly, the anionic analytes chemisorb to the cit-AgNPs only after the silver surface is activated with cations. This leads to the damping of the SPR indicating a chemical coupling between nanoparticle and adsorbate.

Fumaric acid and salicylic acid show the strongest SERS features when the cit-AgNPs are activated with Al^3+^. Here, we suggest that the chemisorption takes place through the carboxy group. In case of uric acid, Pb^2+^ adions provide the highest Raman enhancement, a chemisorption through the NCO^−^ group being proposed.

The chemisorption of the organic acids is enhanced for the anions of higher affinities to the silver surface. More precisely, the following affinity order was observed: citrate < fumaric acid < salicylic acid < uric acid.

## Materials and Methods

**Synthesis of the citrate-capped silver nanoparticles (cit-AgNPs).** All reagents were of analytical grade. Silver colloids were obtained by the common citrate reduction method [38]. Briefly, 0.017 g AgNO_3_ were dissolved in 98 mL ultrapure water under magnetic stirring. After the solution reached the boiling temperature, 2 mL of 1% sodium citrate solution was added and the solution was left to boil for another 30 min. A pH 6 was measured after the colloid synthesis.

**SERS measurements.** For the SERS measurements, the cit-AgNPs were activated with Ca^2+^, Pb^2+^ or Al^3+^ cations at a final concentration of 50 µM, which were added to the colloidal solution in the form of nitrate or sulfate salts: Ca(NO_3_)_2_, Pb(NO_3_)_2_ and Al_2_(SO_4_)_3_. However, similar results were obtained by activating the colloids before or after addition of the analyte.

SERS spectra of the organic acids at a concentration of 50 µM were acquired with a Renishaw InVia Raman spectrometer equipped with a Nd:YAG frequency-doubled laser emitting at 532 nm at a power of ≈20 mW measured on the sample. In form of 10 µL drops, the sample solution was placed on a microscope slide covered with aluminum foil. The laser was focused on the liquid drop using a 5× objective (NA = 0.12). The spectra were recorded by averaging four acquisitions of 4 s exposure each unless otherwise stated.

## Supporting Information

File 1 Additional experimental data.
