# Supplementary material for: The role of Ag+, Ca2+, Pb2+ and Al3+ adions in the SERS turn-on effect of anionic analytes
Source: Beilstein J Nanotechnol. 2019 Nov 27;10:2338–45. doi: 10.3762/bjnano.10.224 (PMC6902780; doi:10.3762/bjnano.10.224)
Supplement: File 1 — Additional experimental data. [file Beilstein_J_Nanotechnol-10-2338-s001.pdf]

BEILSTEIN JOURNAL OF NANOTECHNOLOGY

Supporting Information

for

The role of Ag^{+}, Ca^{2+}, Pb^{2+} and Al^{3+} adions in the SERS turn-on effect of anionic analytes

Stefania D. Iancu, Andrei Stefancu, Vlad Moisoiu, Loredana F. Leopold and Nicolae Leopold

Beilstein J. Nanotechnol. **2019,** *10,* 2338–2345. doi:10.3762/bjnano.10.224

Additional experimental data

License and Terms: This is a supporting information file under the terms of the Creative Commons Attribution License (http://creativecommons.org/licenses/by/4.0). Please note that the reuse, redistribution and reproduction in particular requires that the authors and source are credited.
The license is subject to the *Beilstein Journal of Nanotechnology* terms and conditions: (https://www.beilstein-journals.org/bjnano)

Content

•Additional SERS enhancement of citrate at pH 4 due to Al^{3+} adions

•UV-vis extinction spectra of the colloidal solutions

• Raman and SERS spectra of the organic acids

• SERS spectra of salicylic acid in the 6–9 pH range

• Additional evidence for the role of adions in the SERS turn-on effect

Additional SERS enhancement of citrate at pH 4 due to Al^{3+} adions

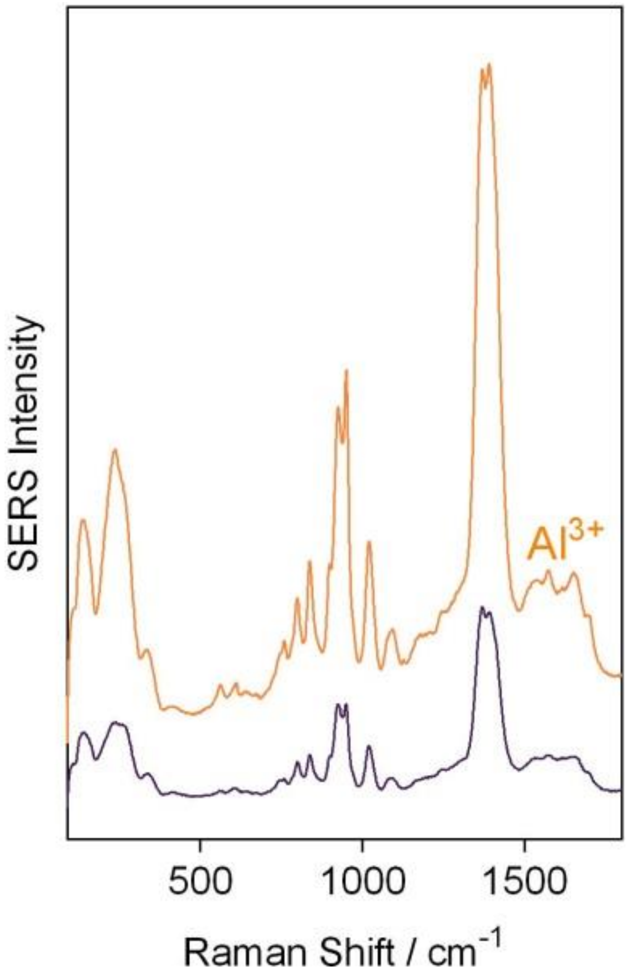

Figure S1. Additional intensity enhancement of the SERS spectrum of citrate at pH 4 in the presence of Al^{3+} adions. From bottom to top: SERS spectrum of citrate obtained from cit-AgNPs at pH 4 (pH lowering by addition of HNO_3) and SERS spectrum of citrate obtained from cit-AgNPs at pH 4 supplemented with Al^{3+} 50 µM (added in form of sulphate salt $Al_2(SO_4)_3$).

UV-vis extinction spectra of the colloidal solutions

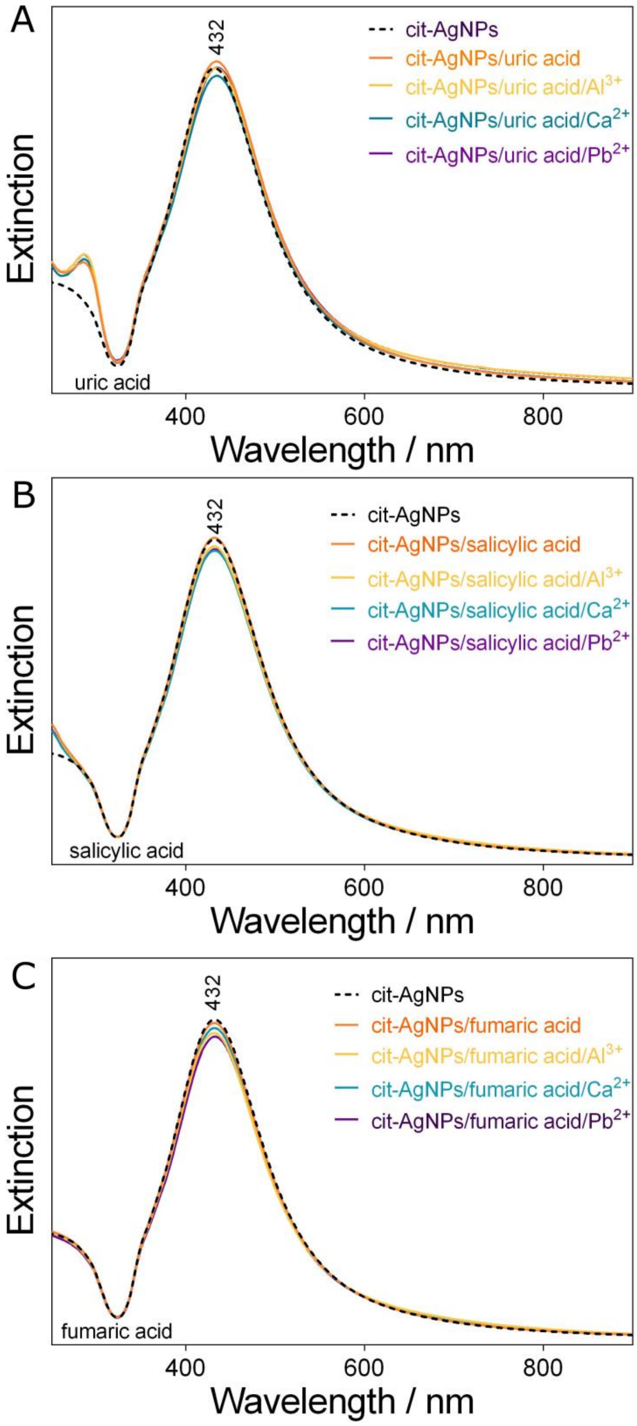

Figure S2. UV-vis extinction spectra of cit-AgNPs recorded at pH 6 and of the mixtures cit-AgNPs/uric acid (A), cit-AgNPs/salicylic acid (B), cit-AgNPs/fumaric acid (C) as well as the UV-vis spectra of the SERS activated cit-AgNPs/organic acid/cation mixtures as indicated in the figure. The final concentration of each organic acid in the solution was 50 µM and that of the Al^{3+}, Ca^{2+}, Pb^{2+} cations 50 µM.

Raman and SERS spectra of the organic acids

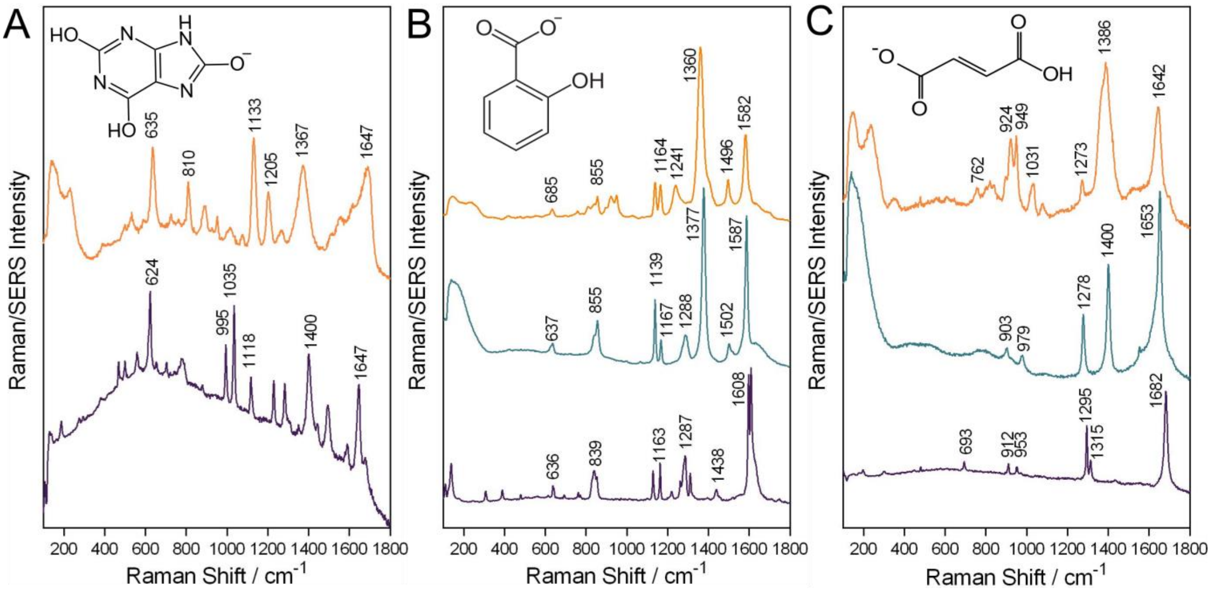

Figure S3. (A) Raman spectrum of powder uric acid and SERS spectrum of uric acid, (B) Raman spectrum of salicylic acid in powder form and in aqueous solution 0.1 M and SERS spectrum of salicylic acid, (C) Raman spectrum of fumaric acid in powder form and in aqueous solution 0.1 M and SERS spectrum of fumaric acid.

The SERS spectra were obtained after the addition of Al^{3+} 50 µM to the cit-AgNPs. For Raman measurements aqueous solutions of salicylic and fumaric acid were obtained by solving the powders in ultrapure water with addition of NaOH in order to ensure a basic pH. All spectra were acquired with a 532 nm laser line.

SERS spectra of salicylic acid in the 6–9 pH range

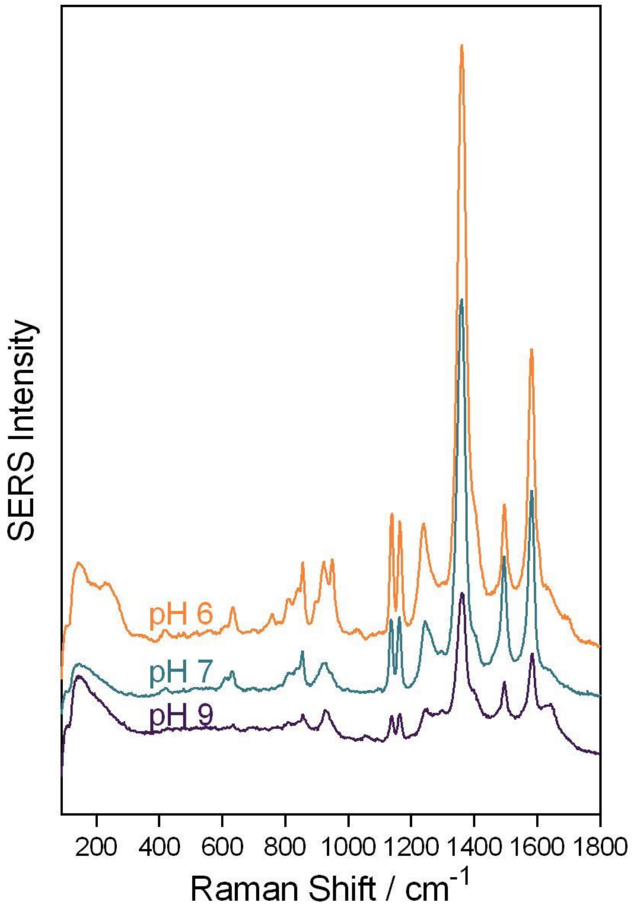

Figure S4. SERS spectra of 50 µM salicylic acid obtained by using Al^{3+} 50 µM activated cit-AgNPs at pH 6, pH 7 and pH 9 as indicated in the figure.

Additional evidence for the role of adions in the SERS turn-on effect

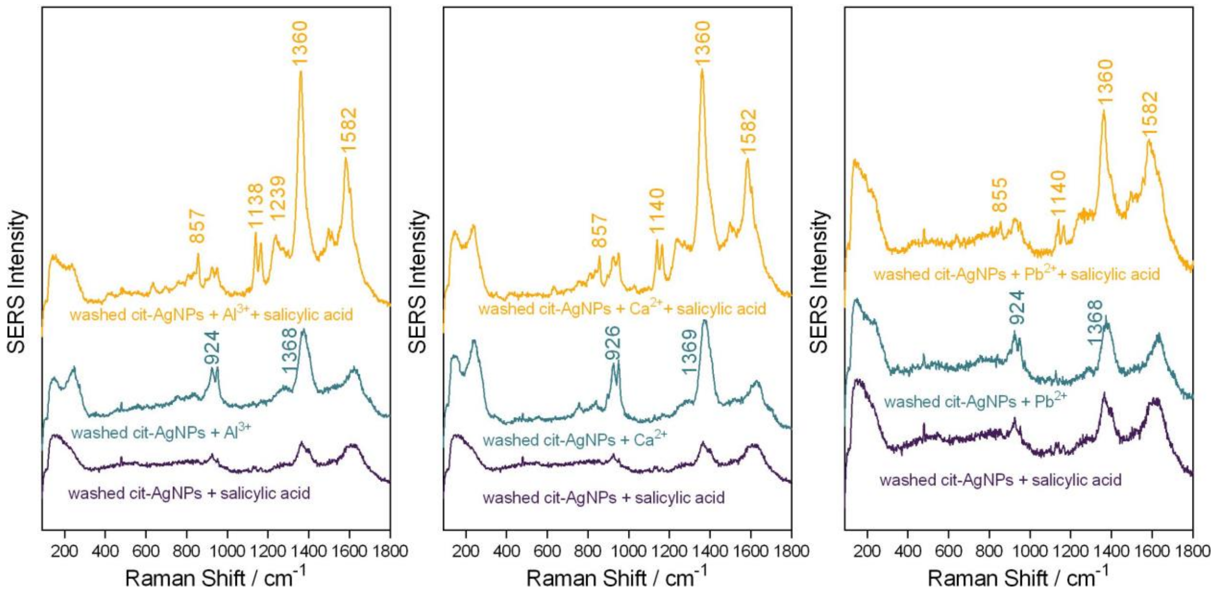

Figure S5. SERS spectra obtained from washed inactivated cit-AgNPs (violet spectra) containing salicylic acid 50 µM, from activated cit-AgNPs with 50 µM Al^{3+}, Ca^{2+} or Pb^{2+} (green spectra, from left to right) and from activated cit-AgNPs with 50 µM Al^{3+}, Ca^{2+} or Pb^{2+} containing salicylic acid 50 µM (yellow spectra, from left to right).

Ca^{2+}, Pb^{2+} or Al^{3+} activated cit-AgNPs were washed by centrifugation and resuspendation in order to eliminate the excess of free, unadsorbed Ca^{2+}, Pb^{2+} or Al^{3+} from the colloidal solution.

For this, Ca^{2+}, Pb^{2+} or Al^{3+} (50 µM final concentration) were added to 1 mL cit-AgNPs and the solution was vortexed for 1 minute to homogenize the solution and ensure the complete adsorption of the cations to the silver surface. Next, the solutions were centrifuged at 5800 g for 10 minutes, the supernatant was removed by pipetting and the cit-AgNPs were resuspended in 1 mL ultrapure water, so that the AgNPs concentration remained the same. By doing so, only the adsorbed cationic adions remain on the surface of the cit-AgNPs, whereas the free cations present in the solution were discarded.

As a reference, inactivated, as synthesized cit-AgNPs underwent the same washing process.

Salicylic acid (50 µM final concentration) was added to both, activated and not activated washed colloidal solutions. The SERS spectra presented in Figure S5 show

that only the Ca^{2+}, Pb^{2+} or Al^{3+}-activated cit-AgNPs enabled the obtaining of the SERS signal of salicylic acid, whereas by using the washed colloidal solution without any cations added resulted in a blank SERS signal of the colloidal solution. However, very weak intensity bands of citrate were observed in the SERS spectra of inactivated cit-AgNPs due to citrate residues remaining on the silver surface adsorbed to Ag^{+} active sites.

The citrate SERS spectrum is clearly observed when recording the SERS spectrum of the Ca^{2+}, Pb^{2+} or Al^{3+} activated cit-AgNPs indicating that after the washing process, the adsorbed cationic adions remain on the surface of the cit-AgNPs together with a considerable amount of citrate capping agent.
